# Chronopharmacology-Driven Precision Therapies for Time-Optimized Cardiometabolic Disease Management

**DOI:** 10.3390/biology15030241

**Published:** 2026-01-28

**Authors:** Shakta Mani Satyam, Sainath Prabhakar, Mohamed El-Tanani, Bhoomendra Bhongade, Adil Farooq Wali, Imran Rashid Rangraze, Ismail Ibrahim Ali Matalka, Yahia El-Tanani, Manfredi Rizzo, Sorina Ispas, Ioannis Ilias, Anna Paczkowska, Viviana Maggio, Karolina Hoffmann

**Affiliations:** 1 Department of Pharmacology, RAK College of Medical Sciences, RAK Medical and Health Sciences University, Ras Al Khaimah 11172, United Arab Emirates; 2 Department of Perfusion Technology, Manipal College of Health Professions, Manipal Academy of Higher Education, Manipal 576104, Karnataka, India; sainath.p@manipal.edu; 3 RAK College of Pharmacy, RAK Medical and Health Sciences University, Ras Al Khaimah 11172, United Arab Emirates; 4 Department of Internal Medicine, RAK College of Medical Sciences, RAK Medical and Health Sciences University, Ras Al Khaimah 11172, United Arab Emirates; 5 Department of Pathology, RAK College of Medical Sciences, RAK Medical and Health Sciences University, Ras Al Khaimah 11172, United Arab Emirates; 6 Royal Cornwall Hospital Trust, National Health Service (NHS), Truro TR1 3LJ, UK; 7 Department of Health Promotion Sciences, Maternal and Infant Care, Internal Medicine and Medical Specialties (PROMISE), School of Medicine, University of Palermo, 90127 Palermo, Italy; 8 Department of Anatomy, Faculty of Medicine, Ovidius University, 900470 Constanta, Romania; 9 Department of Endocrinology, Hippokration Hospital, 11527 Athens, Greece; 10 Department of Pharmacoeconomics and Social Pharmacy, Poznan University of Medical Sciences, 61-701 Poznań, Poland; 11 Department and Clinic of Internal Diseases and Metabolic Disorders, Poznan University of Medical Sciences, 61-701 Poznań, Poland

**Keywords:** chronopharmacology, chronotherapy, cardiometabolic diseases, circadian rhythms, time-optimized therapeutics, hypertension, type 2 diabetes mellitus, precision medicine

## Abstract

Cardiometabolic diseases, including hypertension, type 2 diabetes, dyslipidemia, and obesity, along with their cardiovascular complications, remain leading causes of morbidity and mortality worldwide, imposing significant public health, economic, and societal burdens. Conventional pharmacological therapies often show limited efficacy and increased adverse effects because they do not account for the body’s intrinsic circadian rhythms, which regulate organ function, drug absorption, and metabolism. Chronopharmacology, which aligns treatment timing with these biological rhythms, offers a strategy to enhance therapeutic outcomes. This review presents a comprehensive analysis of chronopharmacology principles applied to cardiometabolic disease management, integrating molecular, physiological, and clinical perspectives. It examines how core clock genes and tissue-specific circadian patterns influence drug action and absorption and summarizes evidence-based time-optimized interventions for hypertension, diabetes, dyslipidemia, obesity, and multimorbid patients. Furthermore, the review highlights emerging innovations, including artificial intelligence-guided dosing, circadian-biomarker-informed therapy selection, and wearable digital devices for real-time monitoring of biological rhythms. By synthesizing mechanistic and clinical insights, circadian-aligned treatment strategies are shown to improve drug efficacy, reduce adverse effects, and support the development of precision, rhythm-based therapeutics, offering a practical framework for personalized cardiometabolic disease care.

## Simple Summary

Cardiometabolic diseases such as high blood pressure, type 2 diabetes, high cholesterol, and obesity are leading causes of illness and death worldwide, affecting millions and straining healthcare systems. Traditional drug treatments often work inconsistently or cause side effects because they ignore the body’s natural daily rhythms, which control how organs function and how medications act. Chronopharmacology is an emerging approach that times treatments to these biological rhythms, making therapies more effective and safer. This review explores how circadian variations in gene expression and tissue function influence drug absorption and therapeutic responses, and how optimizing medication timing can improve clinical outcomes in patients with single or multiple cardiometabolic conditions. It also highlights innovative tools, including artificial intelligence, rhythm-guided biomarkers, and wearable devices, that help tailor treatments to individual biological clocks. By aligning therapies with the body’s natural cycles, chronopharmacology can enhance treatment effectiveness, reduce side effects, and pave the way for precision medicine that improves patient health and quality of life.

## 1. Introduction

Cardiometabolic diseases—including hypertension, type 2 diabetes mellitus, obesity, dyslipidemia, and cardiovascular disorders—represent a tightly interconnected cluster of chronic conditions contributing to over one-third of global mortality and imposing severe economic and societal burdens that are projected to rise significantly in the coming decades [1]. Since 1975, the global prevalence of obesity has nearly tripled, and obesity now affects approximately 650 million adults worldwide, many of whom are at increased risk of developing type 2 diabetes and dyslipidemia. [2,3]. The worldwide prevalence of hypertension exceeds 1.2 billion people, and type 2 diabetes affects more than 500 million people worldwide [4,5]. Current pharmacotherapy and lifestyle interventions have shown progress, but numerous patients remain unable to reach their target values, which demonstrates the restricted capabilities of standard treatment methods and the requirement for individualized and advanced therapeutic solutions.

A key contributor to therapeutic variability is the insufficient integration of circadian biology into clinical practice. The human body operates through self-sustaining 24 h circadian rhythms that regulate cardiovascular function, metabolic pathways, endocrine signals, behavioral cycles, blood pressure patterns, glucose homeostasis, lipid turnover, and hormonal secretion [6,7]. These rhythms arise from transcription–translation feedback loops involving core clock genes, such as *BMAL1*, *CLOCK*, *PER1–3*, *CRY1–2*, *REV-ERBα*/*β*, and *RORα*/*β*, which generate oscillatory gene expression across peripheral organs, including the liver, pancreas, adipose tissue, vasculature, and heart [8,9]. The suprachiasmatic nucleus (SCN) in the hypothalamus acts as the central pacemaker, synchronizing peripheral clocks through light–dark cues, while feeding behavior, hormonal pulses, and physical activity serve as additional entraining signals that fine-tune tissue-specific metabolic rhythms [10].

Disruption of circadian homeostasis caused by shift work, sleep loss, irregular eating schedules, artificial light exposure, or clock gene polymorphisms has been strongly linked to hypertension, insulin resistance, dyslipidemia, obesity, inflammation, and cardiovascular disease. Such circadian misalignment contributes to early-morning blood pressure surges, impaired glucose tolerance, altered lipid metabolism, and endothelial dysfunction, all of which worsen clinical outcomes [11,12,13]. When pharmacotherapy is administered without considering these time-dependent biological patterns, it may lead to reduced efficacy, increased side effects, and inconsistent therapeutic responses.

Emerging preclinical and translational evidence indicates that cardiometabolic risk and therapeutic responsiveness are profoundly influenced by systemic metabolic stress, oxidative imbalance, inflammatory burden, and tissue-specific vulnerability, as demonstrated across models of dyslipidemia, diabetes-associated complications, drug-induced organ toxicity, impaired wound healing, hematological dysfunction, and hormonally driven cardiometabolic risk states [14,15,16,17,18,19,20,21]. Furthermore, lifestyle-dependent metabolic regulation and cellular adaptability, including stress-responsive pathways and stem-cell-driven plasticity, have been increasingly recognized as determinants of disease progression, treatment resistance, and long-term mortality, reinforcing the need for integrated, mechanism-guided, and temporally optimized therapeutic strategies in cardiometabolic disorders [22,23].

Chronopharmacology addresses these limitations by aligning medication timing with endogenous circadian rhythms, thereby improving treatment precision and minimizing adverse outcomes. However, the existing chronotherapy literature remains fragmented, often focusing on isolated preclinical findings or small clinical trials without integrating molecular insights, organ-specific rhythms, or translational clinical evidence into a unified therapeutic framework.

The novelty of this review lies in its comprehensive synthesis of molecular, physiological, and clinical data to define a cohesive chronopharmacology-driven precision therapy model for cardiometabolic diseases. It further evaluates three emerging therapeutic directions—clock-targeted drugs, circadian-biomarker-guided therapy, and AI-enabled adaptive dosing—highlighting their translational potential and implementation pathways. By consolidating mechanistic knowledge with clinical applicability, this review aims to provide a structured roadmap for time-optimized, personalized cardiometabolic disease management and identify key areas for future research.

## 2. Literature Search Strategy

This narrative review was conducted using a structured but non-systematic literature search to synthesize mechanistic, translational, and clinical evidence relevant to chronopharmacology in cardiometabolic diseases. Electronic databases, including PubMed, Scopus, and Web of Science, were searched for peer-reviewed articles published primarily over the past two decades, with selective inclusion of earlier landmark studies where relevant. Search terms included combinations of keywords related to circadian biology, chronopharmacology, chronotherapy, cardiometabolic diseases, hypertension, diabetes mellitus, dyslipidemia, obesity, drug timing, clock genes, circadian biomarkers, wearable monitoring, and artificial intelligence-guided dosing. Studies were included if they provided mechanistic insights, observational associations, or interventional data relevant to time-dependent drug effects on cardiometabolic disorders. Both preclinical and human studies were considered to allow for integration of molecular and clinical perspectives. Articles focusing exclusively on unrelated disease areas or lacking relevance to pharmacological timing were excluded. Given the narrative nature of this review, formal risk-of-bias assessment was not performed; however, the strengths and limitations of available evidence are critically discussed, with particular attention to study design, reproducibility, and clinical applicability.

## 3. Historical Perspective on Chronopharmacology

The first research findings about biological rhythms affecting cardiovascular control systems established the basis for contemporary chronopharmacology [24]. Research using circadian gene knockout mice showed that *CLOCK*, *BMAL1*, *PER*, and *CRY* gene disruptions resulted in hypertension and metabolic problems, including glucose issues, lipid imbalances, and weight gain [25,26]. The research established that the circadian system plays a crucial role in managing cardiometabolic functions through its regulatory mechanisms.

The discovery of core clock genes in *Drosophila melanogaster* led scientists to identify mammalian equivalents, which transformed our knowledge about circadian regulation mechanisms [27,28]. The transcription factors *CLOCK* and *BMAL1* function as key regulators of downstream gene expression, while PER and CRY proteins create a feedback loop that maintains temporal stability [29,30]. The nuclear receptors REV-ERBα/β and RORα/β demonstrated their ability to control circadian patterns through amplitude regulation, phase maintenance, and tissue-specific expression [31]. The discovery of molecular mechanisms behind drug-timing effects enabled researchers to develop new approaches to treating cardiometabolic diseases.

The field of chronopharmacology gained substantial clinical attention through large prospective studies such as the MAPEC and HYGIA trials, which reported improved nocturnal blood pressure control and reduced cardiovascular events when antihypertensive medications were administered at bedtime [32,33]. These studies played an important role in stimulating interest in time-dependent antihypertensive therapy and highlighted the potential relevance of circadian rhythms in cardiovascular risk modulation. However, subsequent critical evaluations have raised concerns regarding methodological aspects of these trials, including study design, reporting transparency, and reproducibility. As a result, their findings have not been uniformly adopted into international hypertension guidelines, and the generalizability of bedtime dosing across diverse patient populations remains uncertain. More recent large-scale randomized evidence has challenged the generalizability of bedtime dosing [34]. The Treatment in Morning versus Evening (TIME) trial, a pragmatic randomized controlled study involving over 24,610 participants, found no significant difference in major cardiovascular outcomes between morning and evening dosing of antihypertensive medications [35]. Importantly, the TIME trial demonstrated that routine evening dosing did not confer additional cardiovascular protection, underscoring the heterogeneity of chronotherapy effects. Collectively, these findings suggest that while antihypertensive chronotherapy may benefit selected patient subgroups, bedtime dosing should not be considered universally applicable. Individual patient characteristics, comorbidities, medication pharmacokinetics, and risk of nocturnal hypotension must be carefully considered. The historical development of chronopharmacology shows how scientists moved from basic observations to molecular discoveries and then to practical medical applications, which now support modern precision medicine approaches that use circadian rhythms.

## 4. Circadian Disruptions in Modern Society

The modern way of living has brought about various disruptive elements that interfere with natural body cycles and lead to an increase in cardiometabolic disease cases. Research shows that artificial light exposure, shift work, and urbanization patterns disrupt the body’s natural circadian rhythm [36,37]. Night-shift workers develop insulin resistance, obesity, dyslipidemia, and hypertension because their sleep–wake cycles, hormonal rhythms, and metabolic processes remain continuously disrupted [37]. The conflict between natural body rhythms and required social timing schedules results in social jet lag, which makes metabolic problems worse.

Irregular feeding patterns, excessive screen time, and insufficient physical activity are behavioral factors that contribute to circadian desynchrony. Research indicates that when people consume nutrients, their body’s peripheral clocks in the liver, adipose tissue, and pancreas become synchronized, which controls glucose and lipid metabolism [38,39,40]. Sleep restriction, together with poor sleep quality, causes disruptions in nighttime hormone release, which affects cortisol, melatonin, and growth hormone production and results in insulin resistance and damaged blood vessel function [41].

Genetic predisposition acts as a factor that determines how much a person is affected by disruptions to their natural circadian rhythms. The *CLOCK*, *PER*, and *CRY* genes contain genetic variations that establish individual sleep–wake preferences and metabolic patterns, but certain genetic changes increase the likelihood of developing obesity, diabetes, and cardiovascular disease [42,43]. The biological link between circadian rhythm disorders and cardiometabolic diseases develops from circadian gene expression disturbances, which impact sympathetic nervous system operation, endothelial function, inflammatory pathways, and mitochondrial metabolic processes [44,45].

The elderly population, along with adolescents and night-shift workers, experiences elevated risk levels [46]. The aging process leads to changes in circadian rhythm patterns, which result in sleep disturbances, metabolic problems, and elevated cardiovascular disease risk [47]. The natural body rhythms of teenagers with delayed chronotypes lose their synchronization with their social duties, which leads to higher chances of developing obesity and insulin resistance. The identification of these factors demonstrates the necessity for coordination of chronopharmacological and lifestyle interventions to minimize the adverse effects of circadian rhythm disturbances.

Table 1 demonstrates how circadian disruption from shift work, sleep deprivation, and irregular feeding patterns causes organ-specific rhythm disturbances that activate multiple cardiometabolic disorders. The heart operates through a normal cycle of sympathetic dominance during the daytime, followed by parasympathetic recovery during nighttime [48]. The heart experiences abnormal rhythm patterns because of chronic disruptions, which cause increased sympathetic activity and decreased heart rate variability and result in early-morning heart attacks and arrhythmias [49]. The liver experiences disrupted circadian rhythms, which cause gluconeogenic and lipid metabolic enzymes to lose their rhythmic patterns, leading to insulin resistance and dyslipidemia [50]. The pancreatic β-cells lose their ability to sense time through *PER2* and *REV-ERBα*, which disrupts their normal insulin secretion pattern and results in elevated blood sugar levels [51]. The disruption of circadian rhythms in adipose tissue causes irregular patterns of leptin and adiponectin, which leads to increased visceral fat storage, body inflammation, and obesity development [52]. The reduced availability of nocturnal nitric oxide leads to vascular endothelial dysfunction, which worsens hypertension and accelerates atherosclerosis development [53,54]. The combination of late-night eating, nocturnal screen use, and shift work creates persistent disruptions to the body’s natural circadian rhythms, which intensify the risk of developing cardiometabolic diseases. Knowledge of organ-specific rhythms enables healthcare providers to develop time-sensitive therapeutic approaches that match natural bodily cycles for creating targeted chronotherapeutic interventions.

## 5. Circadian Biology and Cardiometabolic Regulation

For clarity, the evidence discussed in the following sections is categorized according to study type. Preclinical studies primarily elucidate molecular and physiological mechanisms of circadian regulation. Observational studies describe associations between circadian disruption and cardiometabolic risk. Interventional studies, including randomized controlled trials, evaluate the clinical impact of time-specific drug administration. This distinction is maintained throughout to reflect differences in evidence strength and translational readiness.

The body’s natural time-keeping system is essential for maintaining cardiovascular stability and metabolic balance. Central and peripheral clocks operate in a coordinated manner to regulate cellular metabolism, endocrine function, vascular tone, cardiac performance, and inflammatory responses [59]. The core clock genes *CLOCK*, *BMAL1*, *PER1–3*, and *CRY1–2* generate rhythmic oscillations through transcriptional–translational feedback loops, producing thousands of clock-controlled genes (CCGs) that orchestrate daily metabolic and cardiovascular processes across tissues [60]. CCGs operate through specific tissue mechanisms to control enzymatic pathways, hormone release, and receptor responsiveness, which enables proper daily process execution. Nuclear receptors REV-ERBα/β and RORα/β further refine these rhythms, contributing to tissue-specific metabolic precision [61,62].

**Figure 1:**
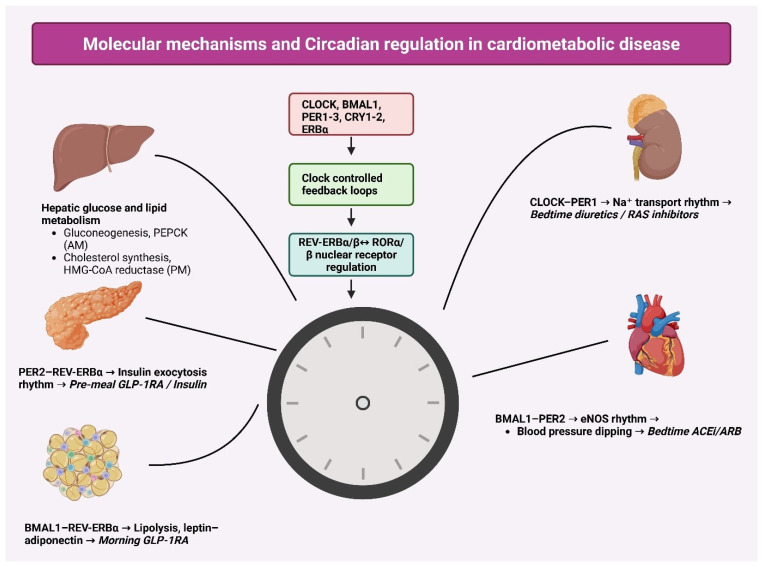
Molecular mechanisms and circadian regulation underlying cardiometabolic disease.

The integrated role of circadian clock machinery in regulating cardiometabolic physiology across major organs is summarized in Figure 1, highlighting how temporal rhythms influence both disease mechanisms and therapeutic timing.

This figure illustrates the core circadian clock components (*CLOCK*, *BMAL1*, *PER*, *CRY*, and REV-ERB/ROR nuclear receptors) and their feedback regulation of metabolic processes relevant to cardiometabolic disease. Key organ-specific rhythms include hepatic glucose/lipid metabolism, pancreatic insulin exocytosis, adipose lipolysis and adipokine release, renal sodium transport, and endothelial nitric oxide signaling. These oscillatory pathways influence optimal timing of therapies such as GLP-1 receptor agonists, insulin, diuretics, and ACEi/ARB medications. This figure was created using BioRender (BioRender.com) under the authorized personal academic license of one of the co-authors, ensuring compliance with BioRender’s publication and licensing policies.

Pharmacokinetic and pharmacodynamic processes also show strong circadian variation. Daily oscillations in hepatic CYP450 enzymes (CYP3A4, CYP2C9, CYP2D6), renal excretion, gastrointestinal motility, and plasma protein binding influence drug absorption, metabolism, clearance, and toxicity [63,64]. Receptor sensitivity and downstream signaling exhibit time-dependent fluctuations, affecting therapeutic efficacy across drug classes. Failure to accommodate these patterns contributes to suboptimal responses, greater adverse effects, and potential drug resistance [65]. Circadian regulation thus exerts integrated control over cardiovascular and metabolic homeostasis throughout the 24 h cycle. Table 2 summarizes key time-dependent physiological fluctuations, including blood pressure, glucose output, lipid metabolism, insulin activity, renal clearance, and adipose function, and their implications for medication timing.

## 6. Tissue-Specific Chronopharmacology and Translational Insights

### 6.1. Cardiac Tissue

The intrinsic circadian system within cardiomyocytes controls how the heart functions through its regulation of contractility, electrophysiology, metabolism, and stress response mechanisms. The cellular oscillators within cardiac tissue actively control the timing of myocardial energy consumption, ion channel operation, and calcium management, which determines both heart output and rhythm stability during the complete 24 h cycle [71]. Research shows that the natural circadian patterns of cardiac function produce daily changes in heart rate and blood pressure and make the heart more prone to arrhythmias [72]. The body experiences its highest heart rate during early morning hours because of increased sympathetic nervous system activity, which leads to elevated risks of heart attacks and sudden cardiac deaths. The heart rate decreases during sleep time, which enables the cardiovascular system to rest [49].

The natural rhythms of the body strongly affect how medical treatments work in cardiology. Beta-blockers to control heart rate and blood pressure work best in the early morning because they decrease myocardial oxygen consumption and block excessive sympathetic nervous system activation [73,74]. The therapeutic effects of calcium channel blockers and ACE inhibitors reach their peak when patients take these medications at times when their heart activity reaches its highest points [75]. Research findings demonstrate their direct application to medical practice. The combination of beta-blockers and ACE inhibitors given at specific times to hypertensive patients leads to fewer cardiovascular events during early morning hours, better blood pressure control at night, and superior long-term heart health results.

### 6.2. Vascular Endothelium

The vascular endothelium operates as a dynamic tissue that depends on circadian clock systems for its complete functional control. The endothelial cells contain the clock genes *BMAL1, PER2*, and *REV-ERBα*, which control the production of nitric oxide, inflammatory responses, vascular tone, and angiogenic processes [76]. The body produces its highest amounts of nitric oxide during the late night and early morning hours, which matches the optimal time for vascular relaxation and maximum blood vessel flexibility [77]. The disruption of normal circadian rhythms by work shifts and sleep disorders decreases endothelial nitric oxide production while creating conditions for hypertension, atherosclerosis, and thromboembolic events [78].

Medical treatments can use the natural time-dependent patterns of the body to delivery better results for cardiovascular patients. The nighttime administration of ACE inhibitors and ARBs matches the natural peak of renin–angiotensin system activity, which helps patients achieve normal blood pressure patterns during sleep and decreases their risk of cardiovascular events. Pharmacological agents that target endothelial circadian pathways through REV-ERBα agonists and PER2 enhancers show promise for improving vascular health through their ability to decrease endothelial inflammation, enhance nitric oxide availability, and reduce vascular stiffness [79,80].

### 6.3. Hepatic Tissue

The liver functions as a primary metabolic center for glucose and lipids, while its operations follow strict control from internal biological rhythms. The metabolic processes of hepatocytes follow natural patterns that result in daily peaks of gluconeogenesis, glycogen synthesis, cholesterol production, and fatty acid breakdown. One of the preclinical studies reported that hepatic oscillators modulate gluconeogenesis, glycogen storage, cholesterol synthesis, and lipid handling [81]. Phosphoenolpyruvate Carboxykinase (PEPCK) activity peaks in the early morning, aligning with fasting-state glucose requirements, whereas 3-Hydroxy-3-Methylglutaryl-Coenzyme A (HMG-CoA) reductase shows the highest activity at night, supporting evening dosing of statins [82,83].

The disruption of hepatic circadian rhythms in people with irregular sleep patterns, shift work, and metabolic disorders results in poor glucose control, lipid disorders, and an elevated risk of non-alcoholic fatty liver disease [84]. One of the preclinical studies reported that taking metformin in the morning matches the natural metabolic peaks of the liver to produce better glucose control and insulin sensitivity [85]. The development of clock-targeted therapeutics, including REV-ERBα agonists, shows promising preclinical results for treating metabolic liver disease through their ability to enhance insulin sensitivity, reduce lipid accumulation, and control inflammatory responses [59].

### 6.4. Pancreatic Islets

The pancreatic β-cells operate with their own internal time-keeping system, which controls insulin production and release and glucose sensitivity. The clock genes *BMAL1* and *PER2* work together to control insulin exocytosis, β-cell calcium signaling, and β-cell number maintenance [86]. The disruption of β-cell circadian rhythms through environmental changes, genetic factors, or aging causes insulin secretion problems, which result in elevated blood sugar after meals, insulin resistance, and type 2 diabetes development [87].

The practice of chronopharmacology enables healthcare providers to match drug administration with natural β-cell activity patterns. The combination of pre-meal GLP-1 receptor agonists with insulin timing and new medications that affect β-cell clock mechanisms leads to better blood sugar control, reduced hypoglycemia, and better metabolic results [88]. Fixing β-cell circadian rhythms enables the recovery of normal glucose-stimulated insulin secretion, which shows promise for treating diabetes through clock-based therapeutic approaches.

### 6.5. Adipose Tissue

The proper functioning of adipose tissue depends on circadian rhythms to achieve energy balance. The natural cycles of lipolysis, adipokine production, and fatty acid breakdown in adipocytes affect how the body regulates appetite and insulin sensitivity and produces systemic inflammation. The disruption of adipocyte clocks results in increased visceral fat storage, persistent inflammation, and metabolic syndrome development [89]. The combination of pharmacological adipokine treatment with scheduled eating times produces better results for weight management through improved adipose tissue metabolic performance [90].

Research studies show that GLP-1 receptor agonist treatment with time-restricted eating leads to better weight loss results and improved insulin sensitivity and lipid profile outcomes [91,92]. The combination of lifestyle and pharmacological treatments through adipose chronotherapy shows promise for treating obesity and metabolic syndrome because it provides a scientific basis for integrating these approaches [93].

### 6.6. Renal Tissue

The body regulates kidney function through circadian rhythms that control sodium processing, glomerular filtration, and drug elimination. The expression of clock genes in renal tubules controls the activity of transporters and enzymes, which leads to daily fluctuations in blood pressure, electrolyte levels, and drug absorption rates [94]. The administration of renin–angiotensin system inhibitors at bedtime through renal-physiology-targeted chronotherapy enhances drug excretion while lowering blood pressure during nighttime and decreasing the risk of cardiovascular and renal complications [95].

The complex relationship between molecular circadian regulators, organ-specific physiology, and time-directed therapeutic approaches becomes evident in Table 3, which demonstrates how these elements work together to achieve optimal pharmacological results. The intrinsic clock genes *BMAL1, CLOCK, PER*, and *REV-ERBα* within each tissue create unique oscillatory patterns that control periodic transcriptional and metabolic operations. The natural fluctuations in the body create daily patterns of cardiac function, blood vessel control, liver processing, insulin release, fat metabolism, and salt handling, which determine when medications produce their best effects with the least harm.

## 7. Translational Implications

The translational value of circadian-informed therapy for cardiometabolic disease treatment stands out because it establishes a fresh method of rhythm-based precision medicine that goes beyond conventional pharmacological treatments. Circadian rhythms regulate multiple physiological processes, including blood pressure, insulin sensitivity, hepatic glucose production, lipid metabolism, renal excretion, and hormonal secretion. Disruption of these rhythms contributes to cardiometabolic disease development. The use of pharmacological treatments that follow the body’s natural oscillations enables clinicians to improve drug responses while minimizing toxic side effects.

Chronotherapy enables patients to follow their treatment plans better and makes it easier to incorporate their medication into their daily routines. Daily medication routines enable better physiological results and help patients maintain their medication schedules. The combination of bedtime antihypertensives with early-morning metformin and preprandial GLP-1 receptor agonists leads to improved patient outcomes and makes it easier for patients to follow their treatment plans. The reduction in complex multidrug treatment plans helps patients with multiple health conditions and multiple medications to follow their medication schedules better because they experience fewer side effects. Current drug delivery strategies focus primarily on optimizing dosing times for individual medications. Future challenges will involve coordinating the timing of multiple drugs while minimizing adverse interactions. Neutral and negative trials, including the TIME trial and other pragmatic studies, highlight the complexity of translating circadian biology into clinical benefit. These findings emphasize that chronotherapy effects are context-dependent and reinforce the need for individualized approaches rather than universal dosing recommendations.

Research now focuses on identifying dependable circadian biomarkers for personalized drug delivery and developing wearable technology platforms with digital health systems for ongoing patient monitoring and predictive algorithms that modify treatment plans in real time during active patient care [104]. Machine learning methods enable researchers to determine the most suitable drug administration times for complex multidrug treatments by considering individual differences in circadian rhythms, medical conditions, and drug absorption patterns [105,106].

The management of cardiometabolic disease may experience a complete transformation through circadian-based precision medicine. The practice of temporal personalization enables clinicians to shift away from standard dosing practices toward producing enhanced patient results through individualized care and optimal safety and effectiveness. Medical chronotherapy requires time-dependent drug delivery systems to build a systematic polypharmacy framework that applies rhythm-based methods for better treatment results and improved patient health outcomes. The development of translational research has the potential to make circadian-informed therapy an essential part of modern precision medicine, which provides customized treatment plans based on individual biological requirements for managing complex cardiometabolic multimorbidity.

## 8. Emerging Strategies in Digital Health, Artificial Intelligence, Circadian Biomarkers, and Lifestyle Chronotherapy

Recent advances in digital health, artificial intelligence (AI), circadian biomarker science, and lifestyle chronotherapy have transformed the therapeutic landscape of cardiometabolic disease management. Contemporary treatment paradigms increasingly integrate chronopharmacology with digital health technologies, AI-driven analytics, circadian biomarkers, and lifestyle-based timing interventions to move beyond conventional “one-size-fits-all” strategies. Traditional chronotherapy has largely relied on standardized dosing schedules derived from population-level pharmacokinetic and pharmacodynamic data; however, substantial interindividual variability in circadian rhythms—driven by genetic polymorphisms, environmental cues, sleep architecture, occupational patterns, and daily behavioral cycles—necessitates data-driven, personalized therapeutic frameworks.

**Figure 2:**
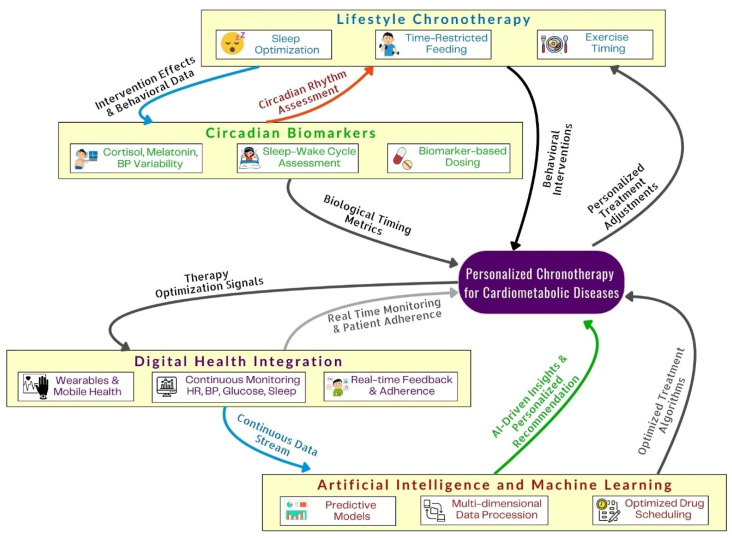
Framework for integrating lifestyle, biomarker, digital, and AI-driven approaches in personalized chronotherapy for cardiometabolic diseases.

The convergence of wearable sensor technologies, continuous physiological monitoring systems, and advanced machine learning algorithms now enables real-time characterization of individual circadian phenotypes and dynamic treatment optimization. These systems allow clinicians to tailor drug selection, dosing, and administration timing according to patient-specific biological rhythms, thereby improving therapeutic efficacy and clinical outcomes [107,108]. A conceptual framework integrating circadian biomarkers, lifestyle timing strategies, digital health tools, and AI-supported clinical decision systems is illustrated in Figure 2.

This figure illustrates a comprehensive model of personalized chronotherapy for cardiometabolic diseases, integrating lifestyle-based timing interventions (sleep optimization, time-restricted feeding, exercise timing), circadian biomarkers, digital health monitoring, and AI-assisted analytics. Continuous physiological and behavioral data from wearables, real-time adherence tracking, and biomarker-guided dosing feed into machine learning algorithms that optimize individual treatment schedules and therapeutic adjustments. This figure was created using BioRender (BioRender.com) under the authorized personal academic license of one of the co-authors, ensuring compliance with BioRender’s publication and licensing policies.

### 8.1. Digital Health Integration

Digital health platforms, including wearable devices and mobile health applications, facilitate continuous, non-invasive monitoring of key physiological parameters, such as heart rate, blood pressure, glucose levels, sleep architecture, physical activity, and energy expenditure [109]. Continuous glucose monitoring (CGM) systems provide real-time glycemic profiles, while ambulatory blood pressure monitors capture diurnal and nocturnal blood pressure variability during routine daily activities. The aggregation of these multimodal data streams into centralized digital platforms enables clinicians to assess not only the magnitude but also the timing, duration, and persistence of circadian disturbances, supporting more precise and responsive therapeutic decision-making.

Importantly, digital health integration allows early identification of high-risk circadian windows, such as exaggerated morning blood pressure surges or postprandial glycemic excursions. For example, detection of early-morning hypertension may guide bedtime administration of ACE inhibitors or angiotensin receptor blockers, while CGM-derived postprandial glucose patterns can inform optimal timing of GLP-1 receptor agonists. Additionally, digital platforms enhance patient engagement through real-time feedback, adherence monitoring, and behavioral reinforcement, all of which are critical for sustained control of chronic cardiometabolic conditions.

### 8.2. Artificial Intelligence and Machine Learning

AI and machine learning algorithms play a pivotal role in analyzing high-dimensional datasets generated from wearable sensors, electronic health records, genomics, metabolomics, and circadian biomarkers. By identifying temporal physiological patterns and complex nonlinear interactions, these algorithms generate individualized predictions of drug response, adverse event risk, and optimal dosing windows. AI-driven models that integrate circadian variables with pharmacogenomic and lifestyle data enable the development of adaptive chronotherapeutic regimens that maximize efficacy while minimizing toxicity.

Clinical and translational studies indicate that AI-guided chronotherapy improves blood pressure stability, enhances glycemic control, and increases medication adherence through dynamically optimized treatment strategies aligned with individual circadian profiles [110]. Predictive machine learning models combining CGM data, sleep–wake cycles, and meal timing have demonstrated the ability to forecast hyperglycemic events, enabling proactive and preventive therapeutic interventions [111]. Furthermore, AI-based scheduling of multidrug regimens optimizes drug combinations and dosing intervals by accounting for circadian pharmacokinetics and pharmacodynamics, thereby reducing drug–drug interactions and cumulative toxicity in patients receiving complex cardiometabolic therapies [112,113].

### 8.3. Circadian Biomarkers

Circadian biomarkers provide objective measures of endogenous biological timing and are central to the implementation of precision chronotherapy. Established biomarkers include cortisol secretion rhythms, melatonin profiles, diurnal blood pressure variation, heart rate variability, core body temperature oscillations, and timed glucose fluctuations. Assessment of hypothalamic–pituitary–adrenal axis activity through salivary or plasma cortisol, along with melatonin secretion patterns, offers insight into sleep–wake cycle integrity and circadian phase alignment [114].

The incorporation of circadian biomarker assessment enables clinicians to detect circadian misalignment and to individualize therapeutic schedules based on internal biological time rather than external clock time alone. Moreover, longitudinal monitoring of these biomarkers allows dynamic evaluation of treatment response and facilitates ongoing adjustment of therapeutic strategies, supporting a flexible and adaptive model of cardiometabolic care.

### 8.4. Lifestyle Chronotherapy

Lifestyle modification remains a cornerstone of cardiometabolic disease management, with growing evidence that its effectiveness is significantly enhanced when aligned with endogenous circadian rhythms. Optimization of sleep duration and quality improves insulin sensitivity, blood pressure regulation, and endothelial function [115]. Circadian synchronization is further supported by minimizing nighttime exposure to artificial light and maintaining consistent sleep–wake schedules.

Time-restricted feeding, which confines caloric intake to the biologically active phase, reinforces peripheral circadian clocks and improves glucose metabolism, lipid handling, and inflammatory profiles. Studies suggest that individuals with obesity or metabolic dysfunction experience greater weight loss, enhanced insulin sensitivity, and improved cardiovascular outcomes when pharmacotherapy is synchronized with feeding windows [116]. Similarly, the timing of physical activity influences metabolic and cardiovascular benefits, with exercise during the active phase enhancing mitochondrial efficiency, endothelial function, and insulin responsiveness, whereas mistimed activity may attenuate these effects.

### 8.5. Combinatorial Chronotherapy

The most effective chronotherapeutic strategies employ an integrated approach that combines pharmacological treatment with circadian biomarker monitoring, AI-driven decision support, and rhythm-aligned lifestyle interventions. Coordinated scheduling of medications, meals, sleep, and physical activity enhances therapeutic windows while reducing adverse effects. This multimodal framework enables true precision medicine by tailoring interventions to individual circadian profiles while simultaneously addressing multiple cardiometabolic comorbidities.

The future of precision cardiometabolic care will increasingly depend on the integration of digital health infrastructures, AI analytics, circadian biomarkers, and lifestyle chronotherapy [117]. These approaches support biologically synchronized treatment regimens that improve drug efficacy, minimize adverse outcomes, and promote sustained long-term benefits. As summarized in Table 4, the advancement of chronotherapy is driven by the convergence of digital technologies, systems biology, and behavioral science. Continuous wearable monitoring—including ECG-enabled smartwatches, CGMs, and 24 h blood pressure devices—enables real-time assessment of circadian phase and variability [118]. When coupled with AI and machine learning, these systems generate predictive chronoprofiles that identify optimal drug administration windows [119]. Longitudinal data analysis allows forecasting of individualized blood pressure peaks and glycemic vulnerability periods, facilitating adaptive medication scheduling responsive to behavioral and environmental changes. The integration of circadian biomarkers such as melatonin, cortisol, and heart rate variability further refines internal biological time estimation, particularly in individuals with irregular sleep patterns. Finally, lifestyle chronotherapy strategies, including time-restricted eating, scheduled exercise, and light-exposure management, restore circadian alignment and amplify pharmacological efficacy [120]. Collectively, this multidimensional, feedback-driven framework represents a transformative shift toward personalized, rhythm-based cardiometabolic healthcare.

While chronopharmacological principles are increasingly supported by mechanistic and early clinical data, most emerging applications discussed in this section remain investigational, with limited validation from large, multicenter randomized controlled trials. To aid interpretation, the strength and type of evidence supporting chronopharmacological approaches across cardiometabolic conditions are summarized in Table 5.

## 9. Limitations of Current Evidence and Knowledge Gaps

Despite growing interest in chronopharmacology, several limitations constrain its clinical implementation. Much of the supportive evidence remains preclinical or observational, limiting causal inference. Randomized controlled trials evaluating hard clinical endpoints are relatively few, and existing trials demonstrate heterogeneous results across populations and drug classes. Interindividual variability in circadian phases, lifestyle patterns, comorbidities, and medication pharmacokinetics further complicates standardized recommendations. Additionally, many studies rely on surrogate outcomes rather than long-term clinical endpoints. Emerging approaches involving circadian biomarkers, wearable technologies, and artificial intelligence are promising but remain largely investigational, with limited prospective validation. Addressing these limitations through well-designed, multicenter randomized trials and standardized chronobiological assessments will be essential before widespread clinical adoption.

## 10. Conclusions

Chronopharmacology has evolved from a conceptual framework into an evidence-informed therapeutic strategy, demonstrating that drug timing is a biologically meaningful determinant of efficacy, safety, and interindividual variability in cardiometabolic disease management. This review consolidates molecular, physiological, and clinical evidence showing that circadian regulation of cardiovascular function, glucose homeostasis, lipid metabolism, hormonal signaling, and vascular biology produces predictable temporal patterns in disease activity and treatment response across hypertension, type 2 diabetes mellitus, dyslipidemia, obesity, and multimorbid cardiometabolic states. Collectively, the evidence discussed indicates that failure to account for endogenous circadian organization contributes to suboptimal therapeutic outcomes, increased adverse effects, and inconsistent response to standard time-independent pharmacotherapy.

The synthesis presented here further demonstrates that rhythm-aligned interventions supported by clock-controlled molecular pathways, organ-specific circadian dynamics, and clinical chronotherapy data can refine therapeutic windows and enhance treatment precision. Integration of circadian biomarkers enables objective assessment of internal biological time, while digital health platforms and artificial intelligence-driven adaptive dosing facilitate continuous monitoring and real-time optimization of therapy. In parallel, lifestyle chronotherapy, including sleep optimization, time-restricted feeding, and appropriately timed physical activity, emerges as a critical adjunct that reinforces pharmacological efficacy by restoring circadian synchrony at behavioral and metabolic levels.

At the same time, important translational gaps remain. The reviewed literature underscores the need for large-scale, multicenter clinical trials to validate chronotherapeutic strategies across diverse populations, systematic evaluation of chronotherapy in polypharmacy and multimorbidity, and standardized validation of circadian biomarkers for routine clinical use. Addressing these challenges will require coordinated efforts integrating chronobiology, translational pharmacology, clinical trial methodology, and digital health infrastructure.

Overall, this review provides a structured and integrative synthesis establishing chronopharmacology-driven precision therapy as a mechanistically grounded and clinically actionable approach to cardiometabolic disease care. By unifying biological timing, technological innovation, and patient-specific treatment adaptation, it defines a clear pathway toward rhythm-optimized, personalized interventions with the potential to improve long-term cardiometabolic outcomes at both individual and population levels.

## Figures and Tables

**Table 1:** Comparative summary of circadian misalignment and its pathophysiological impact on cardiometabolic organs.

Organ/System	Normal Circadian Function	Disrupted State (Shift Work, Sleep Deprivation, Jet Lag)	Pathophysiological Outcome	Therapeutic Opportunity
Heart [55]	Daytime dominance of sympathetic tone; nocturnal parasympathetic recovery	Persistent sympathetic activation, loss of nocturnal BP dipping	Morning MI, arrhythmias	Bedtime antihypertensive chronotherapy
Liver [56]	Morning gluconeogenesis, nocturnal cholesterol synthesis	Loss of rhythm in glucose/lipid enzymes	Insulin resistance, dyslipidemia	Time-targeted metformin/statin dosing
Pancreas [57]	β-cell insulin secretion peaks during active phase	Flattened insulin rhythm, hyperglycemia	T2DM progression	Pre-meal GLP-1 agonists
Adipose Tissue [58]	Daytime lipolysis, nocturnal adipokine secretion	Visceral fat accumulation, leptin resistance	Obesity, inflammation	Timed feeding, adipokine-targeted drugs
Vascular Endothelium [59]	Nocturnal nitric oxide peak, reduced inflammation	Decreased NO bioavailability, endothelial dysfunction	Hypertension, atherosclerosis	REV-ERB agonists, bedtime RAS blockers

**Table 2:** Circadian regulation of key cardiometabolic drug targets and implications for time-optimized therapy.

Physiological Domain/Target	Core Circadian Rhythm	Molecular Mechanism	Therapeutic Implication	Chrono-Optimized Dosing Window
Blood Pressure and Vascular Tone [66]	Morning surge; nocturnal dip	*BMAL1*, *PER2* regulation of eNOS and sympathetic activity	Bedtime dosing restores dipping patterns and reduces CV events	Evening–bedtime (22:00–23:00) for ACE inhibitors, ARBs
Glucose Metabolism (Liver, Muscle) [67]	Fasting peak glucose in early morning	*CLOCK–BMAL1* modulation of gluconeogenic enzymes (PEPCK, G6Pase)	Synchronizing metformin dosing with hepatic clock enhances fasting glucose control	Early morning (06:00–08:00) for metformin
Insulin Secretion (β-cells) [68]	Peak responsiveness during active phase	*PER2*, *REV-ERBα* influence insulin exocytosis	Aligning GLP-1RA or insulin dosing with β-cell rhythmicity improves postprandial control	Preprandial (before main meals)
Lipid Metabolism (Liver) [69]	Nocturnal cholesterol synthesis	HMG-CoA reductase circadian peak at night	Maximizes LDL-C reduction and minimizes hepatic stress	Night (21:00–23:00) for short-acting statins
Adipose Tissue Metabolism [52]	Daytime peak in lipolysis; nocturnal adipokine secretion	Circadian leptin–adiponectin oscillations	Enhances lipolytic efficacy and insulin sensitivity	Morning (08:00–09:00) for GLP-1RA
Renal Sodium Excretion [70]	Daytime natriuresis; nocturnal retention	*CLOCK–PER* regulation of tubular Na^+^ transporters	Improves blood pressure and nocturnal dipping	Bedtime for diuretics and RAS inhibitors

**Table 3:** Integrated tissue-specific chronopharmacological targets, mechanisms, and time-optimized therapeutic implications.

Tissue/System	Key Clock Genes/Regulators	Major Physiologic Rhythms	Representative Drug/Class	Mechanistic Target/Action	Circadian Peak Phase	Optimal Dosing Window	Chronotherapeutic/Clinical Outcome
Heart [96]	*BMAL1*, *PER2*	Contractility, heart rate, electrophysiologic stability	β-blockers	β_1_-adrenergic blockade to blunt sympathetic surge	Early morning (06:00–10:00)	Morning	Attenuates early-morning BP and HR surges; reduces myocardial infarction and arrhythmia risk
Vascular Endothelium [97,98]	*BMAL1*, *PER2*, *REV-ERBα*	Nitric oxide (NO) synthesis, endothelial relaxation, vascular tone	ACE inhibitors /ARBs	Inhibit RAAS activation and oxidative stress	Night (22:00–02:00)	Bedtime	Restores nocturnal dipping, enhances endothelial function, reduces nocturnal BP and cardiovascular risk
Liver [99,100]	*CLOCK*, *BMAL1*, *REV-ERBα*	Gluconeogenesis, glycogen turnover, cholesterol synthesis	Metformin/statins	AMPK activation; inhibition of HMG-CoA reductase	Dual peaks: gluconeogenesis (04:00–08:00), cholesterol synthesis (00:00–04:00)	Metformin: early morning (04:00–08:00); Statins: late evening (00:00–04:00)	Enhances fasting glucose control and maximizes LDL-C reduction; improves hepatic insulin sensitivity
Pancreas (β-cells) [101]	*PER2*, *BMAL1*	Insulin secretion, β-cell responsiveness to glucose	GLP-1 receptor agonists/insulin	Stimulate insulin release, improve β-cell function	Preprandial periods (06:00–08:00, 12:00–14:00, 18:00–20:00)	Before meals	Optimizes postprandial glycemia, minimizes hypoglycemia risk, enhances satiety
Adipose Tissue [102,103]	*BMAL1*, *REV-ERBα*	Lipolysis, adipokine (leptin, adiponectin) secretion, energy storage	GLP-1 agonists /adipokine modulators	Modulate lipid mobilization and adipokine signaling	Active phase (daytime)	Morning/daytime	Improves insulin sensitivity, reduces visceral fat, promotes weight loss and metabolic homeostasis
Kidney [75]	*PER1*, *BMAL1*	Sodium excretion, GFR, RAAS modulation	ACE inhibitors /ARBs/diuretics	Regulate tubular sodium transport and nocturnal BP	Night (22:00–04:00)	Bedtime	Enhances nocturnal natriuresis, restores dipping BP pattern, protects renal and cardiovascular function

**Table 4:** Integration of AI, biomarkers, and lifestyle in future chronotherapeutic frameworks.

Component	Core Function	Technological or Clinical Example	Chronopharmacological Utility	Expected Clinical Impact
Wearable Monitoring Systems	Continuous tracking of BP, glucose, HR, activity, sleep	Apple Watch ECG, CGM (Dexcom G7), ambulatory BP monitoring	Real-time circadian profiling; detection of morning BP surge or nocturnal hyperglycemia	Personalized dosing and early intervention
AI and Machine Learning Models	Integration of multimodal datasets (genomics + chronome + lifestyle)	Predictive circadian drug response modeling	Optimize multidrug timing, prevent overlap toxicity	Reduced adverse events, improved adherence
Circadian Biomarkers	Physiological markers reflecting circadian phase (melatonin, cortisol, HRV)	Melatonin assay, salivary cortisol index	Identify optimal dosing window and phase shifts	Personalized rhythm-based pharmacotherapy
Lifestyle Chronotherapy	Synchronization of feeding, exercise, and sleep with circadian physiology	Time-restricted eating, morning exercise	Enhances drug efficacy via metabolic entrainment	Weight reduction, metabolic resilience
Combinatorial Chronomedicine	Integration of pharmacologic + behavioral + AI-driven rhythm alignment	AI-guided dosing with lifestyle feedback loops	Comprehensive patient-specific circadian modulation	Paradigm shift to adaptive, precision therapeutics

**Table 5:** Summary of chronopharmacology evidence by study type.

Therapeutic Area	Preclinical Evidence	Observational Studies	Randomized Controlled Trials
Hypertension	Strong	Moderate	Mixed (MAPEC/HYGIA vs. TIME)
Diabetes	Strong	Moderate	Limited
Dyslipidemia	Moderate	Limited	Limited
AI-guided dosing	Emerging	Limited	Lacking

## Data Availability

All data arising from this study are included within the article.

## Abbreviations

The following abbreviations are used in this manuscript:

Abbreviation	Full Form
ACEi	Angiotensin-Converting Enzyme Inhibitor
AI	Artificial Intelligence
AM	Ante Meridiem (morning)
ARB	Angiotensin II Receptor Blocker
BMAL1	Brain and Muscle ARNT-Like 1
BP	Blood Pressure
CCG	Clock-Controlled Gene
CGM	Continuous Glucose Monitoring
CLOCK	Circadian Locomotor Output Cycles Kaput
CRY1–2	Cryptochrome Circadian Regulator 1 and 2
CV	Cardiovascular
CYP	Cytochrome P450 Enzyme
DPP-4	Dipeptidyl Peptidase-4
eNOS	Endothelial Nitric Oxide Synthase
G6Pase	Glucose-6-Phosphatase
GFR	Glomerular Filtration Rate
GLP-1	Glucagon-Like Peptide-1
GLP-1RA	Glucagon-Like Peptide-1 Receptor Agonist
HMG-CoA	3-Hydroxy-3-Methylglutaryl-Coenzyme A
HR	Heart Rate
HRV	Heart Rate Variability
MAPEC	Monitorización Ambulatory para Predicción de Eventos Cardiovasculares (Ambulatory Blood Pressure Monitoring for Prediction of Cardiovascular Events) Trial
MI	Myocardial Infarction
NO	Nitric Oxide
PER1–3	Period Circadian Regulator 1 to 3
PCSK9	Proprotein Convertase Subtilisin/Kexin Type 9
PEPCK	Phosphoenolpyruvate Carboxykinase
PK	Pharmacokinetics
PD	Pharmacodynamics
RAAS	Renin–Angiotensin–Aldosterone System
REV-ERBα/β	Reverse Erythroblastosis Virus Nuclear Receptor Alpha/Beta
RORα/β	Retinoic Acid Receptor-Related Orphan Receptor Alpha/Beta
SCN	Suprachiasmatic Nucleus
SGLT2	Sodium–Glucose Co-Transporter 2
T2DM	Type 2 Diabetes Mellitus
TRF	Time-Restricted Feeding
LDL-C	Low-Density Lipoprotein Cholesterol
HDL-C	High-Density Lipoprotein Cholesterol
AMPK	AMP-Activated Protein Kinase
CGMs	Continuous Glucose Monitors
HYGIA	Hygia Chronotherapy Trial
RAS	Renin–Angiotensin System
β_1_	Beta-1 Adrenergic Receptor
AI-Driven	Artificial Intelligence-Driven
ML	Machine Learning
